# Stanford A aortic dissection 40 years after aortic valve replacement with a Starr–Edwards caged-ball prosthesis: a case report

**DOI:** 10.1093/jscr/rjae707

**Published:** 2024-11-16

**Authors:** Tomislav Tokic, Lea Hasnas, Lovro Mikulic, Pavel Markovic, Stella Gustek, Anamarija Kucina, Ivana Jurca, Dubravka Sipus, Martina Zrno Mihaljevic, Hrvoje Gasparovic, Ivan Burcar

**Affiliations:** Department of Cardiac Surgery, University Hospital Center Zagreb, Kispaticeva 12, 10000 Zagreb, Croatia; Department of Surgery, School of Medicine, University of Zagreb, Salata 3, 10000 Zagreb, Croatia; Department of Surgery, School of Medicine, University of Zagreb, Salata 3, 10000 Zagreb, Croatia; Department of Surgery, School of Medicine, University of Zagreb, Salata 3, 10000 Zagreb, Croatia; Department of Surgery, School of Medicine, University of Zagreb, Salata 3, 10000 Zagreb, Croatia; Department of Surgery, School of Medicine, University of Zagreb, Salata 3, 10000 Zagreb, Croatia; Department of Radiology, University Hospital Center Zagreb, Kispaticeva 12, 10000 Zagreb, Croatia; Department of Cardiology, University Hospital Center Zagreb, Kispaticeva 12, 10000 Zagreb, Croatia; Department of Cardiac Surgery, University Hospital Center Zagreb, Kispaticeva 12, 10000 Zagreb, Croatia; Department of Cardiac Surgery, University Hospital Center Zagreb, Kispaticeva 12, 10000 Zagreb, Croatia; Department of Cardiac Surgery, University Hospital Center Zagreb, Kispaticeva 12, 10000 Zagreb, Croatia

**Keywords:** aortic dissection, bicuspid aortic valve, Stanford A dissection, caged-ball valve, aortic valve replacement

## Abstract

Stanford A aortic dissection is one of the most devastating acute medical conditions due to its high morbidity and mortality. We describe a 77-year-old male patient with a medical history of surgical aortic valve replacement with a still functioning Starr–Edwards caged-ball valve 40 years prior. The patient was promptly diagnosed with an ascending aortic aneurysm and dissection, and an emergency Bentall procedure in deep hypothermic circulatory arrest was performed. This is, to the best of our knowledge, the first Stanford A dissection case described in the literature in a patient with Starr–Edwards valve, and the longest still functioning caged-ball valve to have been replaced with the Bentall procedure. We also discuss the caged-ball valve’s unfavorable hemodynamics as a potential predisposing factor of the dissection, as well as the patient’s supposed initial bicuspid aortic valve disease which could also predispose to aortic aneurysm formation and dissection.

## Introduction

Caged-ball prostheses were among some of the very first mechanical heart valve prostheses to be used to replace diseased heart valves [1]. One of the most prominent types of said valves was the Starr–Edwards valve, which was the fruit of a collaboration between Lowell Edwards, an engineer, and Albert Starr, a surgeon [2]. The first implantation of a Starr–Edwards caged-ball prosthesis was performed in 1960. Since then, said valve has been implanted for decades, and in thousands of patients, with reports about its longevity measuring in decades, and even over half a century [3–5]. However, Starr–Edwards caged-ball prosthesis comes with a number of caveats, namely regarding its thrombogenicity and unnatural blood flow when compared to the native valves, or the more modern prosthetic ones [6]. We present a case of a 77-year-old patient with a Stanford A aortic dissection 40 years after the aortic valve replacement (AVR) with a caged-ball prosthesis. This case marks, to the best of our knowledge, the first Stanford A dissection after the aforementioned prosthesis implantation described in the literature, and also the longest time passed between the Starr–Edwards valve implantation and a Bentall procedure.

## Case report

**Figure 1:**
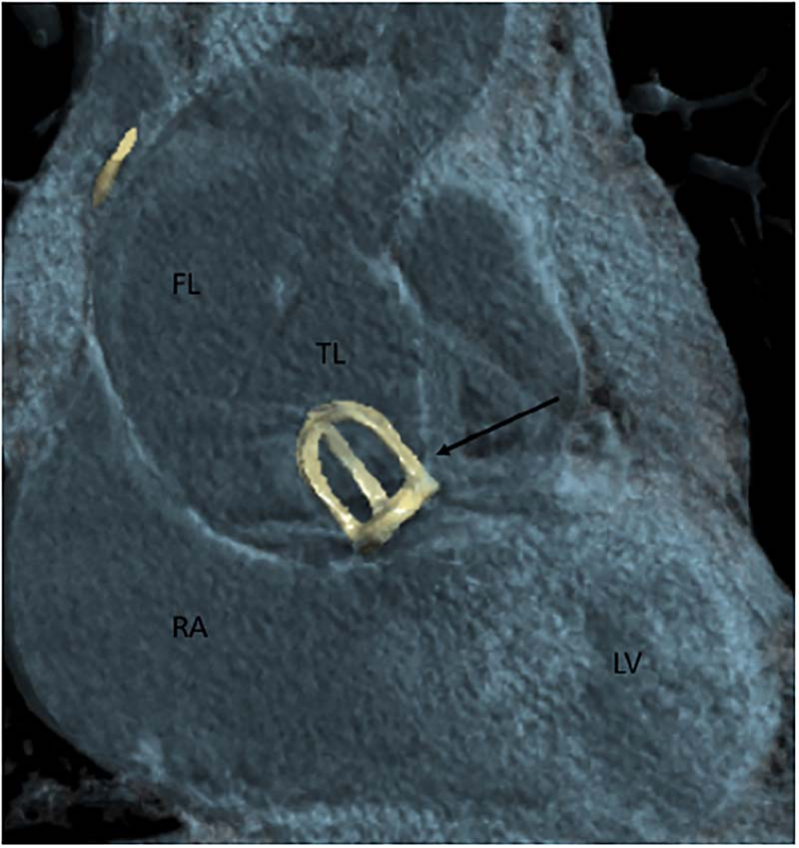
Contrast-enhanced CT aortography coronal plane in volume rendering technique reconstruction. Stanford A dissection distal to artificial caged-ball (thin arrow) mechanical valve. FL, false lumen; TL, true lumen; RA, right atrium; LV, left ventricle.

A 77-year-old male patient presented to the emergency department with chest pain which started a couple of hours before. His medical history is significant of surgical AVR with a mechanical caged-ball prosthesis 40 years prior due to aortic stenosis and atrial fibrillation. Unfortunately, he presented no medical records regarding the previous AVR. Therefore we did not know what was the pathology of the aortic valve at the time. He was anticoagulated with warfarin and at the time of presentation was in a therapeutic range, with an international normalized ration value of 3.01. During the observation in the emergency department, the patient sustained a cardiac arrest with pulseless electrical activity verified on the electrocardiogram. He was successfully resuscitated and promptly transferred to the cardiovascular intensive care unit (CICU). At the time of the CICU admission, the patient had elevated blood lactate levels and was acidotic, and vasopressor support with norepinephrine was initiated. Urgent coronary angiography was performed which showed the absence of coronary artery disease, normal function of the caged-ball prosthesis, and a dilated ascending aorta. Computed tomography (CT) aortography due to the aortic aneurysm was then performed, and the diagnosis of the Stanford A dissection was made. The ascending aorta was up to 60 mm wide, and communication between the false and true lumen was described in a proximal ascending part of the vessel (Fig. 1). Hemopericardium up to 24 mm was also described.

**Figure 2:**
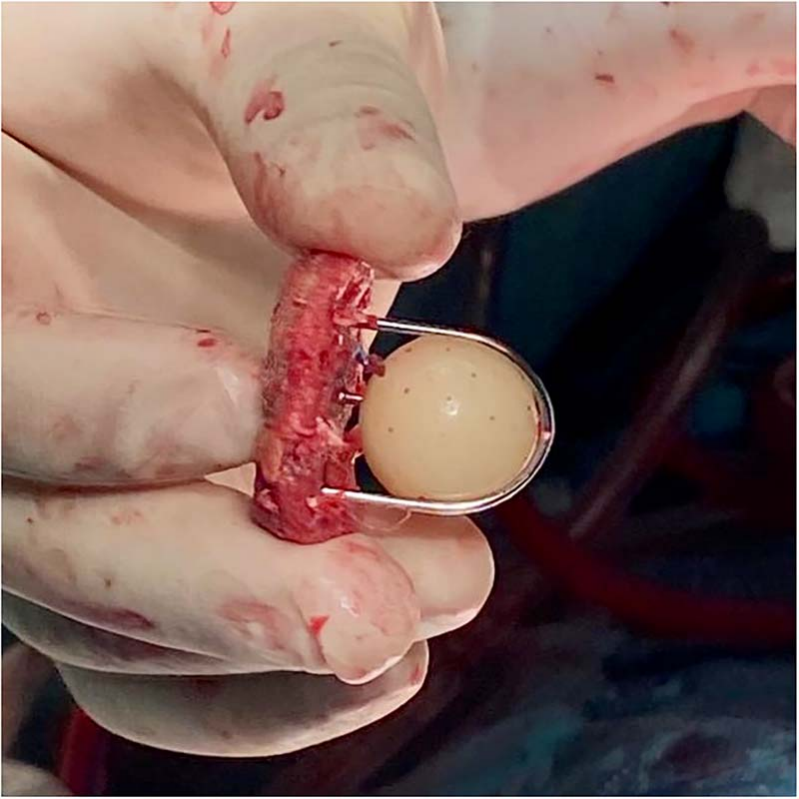
Intraoperative photography showing the excised fully functional Starr–Edwards caged-ball mechanical prosthesis.

The decision was made to perform an emergency cardiac surgical procedure, and the patient was transferred to the operating room. After cannulating the right axillary artery median redo sternotomy was performed, and the operative diagnosis of the dissected ascending aorta was confirmed. The caged-ball aortic prosthesis was visualized after the aortotomy and was pristine, with completely normal mobility of the ball inside the cage. The prosthesis was excised (Fig. 2), and the Bentall procedure in deep hypothermic circulatory arrest was performed. Due to the poor function of the right ventricle, the patient was put on venoarterial extracorporeal membrane oxygenation (ECMO) at the end of the procedure.

The patient was successfully weaned from ECMO 7 days after the Bentall procedure. Unfortunately, the patient passed away 45 days after the initial procedure due to septic shock.

## Discussion

Starr–Edwards mechanical prostheses, with its pioneering caged-ball design, were one of the first prostheses intended to replace diseased native heart valves. The first Starr–Edwards valve was used for mitral valve replacement in 1960, and for AVR shortly after that. The prosthesis has stood the test of time, and it is estimated that more than 175 000 of those prostheses were implanted during the production period until the 2000s [1].

There are a number of reports of patients surviving with functioning Starr–Edwards valves for decades, even more than 50 years after the initial implantation [3–5]. However, since the valve design was one of the earliest forays into the world of artificial heart valves, it departs from natural valves significantly both in form and function [2]. Since the 1960s, there has been a continuous trend to improve artificial heart valve designs to provide better hemodynamics, less thrombogenicity, and more durability. Compared to the more modern heart valve prostheses, the Starr–Edwards valve offers a less natural blood flow which could lead to more turbulent flow and elevated shear stress [6]. Said turbulent blood flow and sheer stress on the aortic wall could further lead to aortic aneurysm formation, which could, in turn, lead to ascending aortic dissection.

It should also be noted that our patient had an AVR performed when he was 37 years old. As he did not present any relevant medical documentation about the procedure, it is unknown what the initial aortic valve pathology was. However, due to the very young age at the time of the AVR, it is likely that he was born with a BAV since the average age for BAV surgery is significantly lower than for tricuspid AVR [7]. While BAV can be found in isolation, it is frequently found in other pathologies, namely ascending aortic dilation. While ascending aortic aneurysm formation in BAV could be explained by pathologic flow dynamics, there are also studies that show structural defects independent of blood flow lesions [8]. Aortic root dilation in BAV has been documented in childhood, which implies the process could begin early. The most lethal complication of BAV and ascending aortic aneurysm is aortic dissection.

Ascending aortic aneurysm and subsequent dissection, therefore, could be, in this patient, both a long-term result of unnatural blood flow through the caged-ball valve or a natural progression of his (supposed) BAV disease. Whatever may be the cause, this case serves as yet another testament to the robustness of the Starr–Edwards design and a cautionary tale about the long-term follow-up of both congenital heart disease patients and those with a history of previous cardiac surgery.
